# Subcutaneous emphysema, pneumomediastinum and pneumoperitoneum after unsuccessful ERCP: a case report

**DOI:** 10.1186/1757-1626-2-120

**Published:** 2009-02-03

**Authors:** Konstantinos Alexiou, Timothy Sakellaridis, Nikolaos Sikalias, Ioannis Karanikas, Nikolaos Economou, Giorgios Antsaklis

**Affiliations:** 1 Department of Surgery, "Sismanoglio" General Hospital of Athens, Athens, Greece

## Abstract

**Background:**

The presence of subcutaneous emphysema, pneumomediastinum and pneumoperitoneum simultaneously is a rare complication of upper gastrointestinal endoscopy that usually indicates free perforation to the peritoneal cavity or the retroperitoneal space.

**Case presentation:**

We report an unusual case of a self-limited subcutaneous emphysema, pneumomediastinum and pneumoperitoneum following an unsuccessful ERCP for removal of a common bile duct stone.

**Conclusion:**

There was no radiological evidence of peritoneal or retroperitoneal perforation. This complication is distinct from pneumomediastinum and pneumoperitoneum due to perforation, and must be recognized, because it is benign and needs no surgical or radiological intervention.

## Background

Endoscopic retrograde cholangiopancreatography (ERCP) is a standard invasive technique for revealing and management of a wide spectrum of distal bile duct disorders. The rate of significant complications is very low if it isn't combined with endoscopic sphincterotomy. Subcutaneous emphysema, pneumomediastinum and pneumoperitoneum after endoscopy indicate free perforation resulting from instrumental trauma. We present a case of subcutaneous emphysema, pneumomediastinum and pneumoperitoneum after ERCP without an obvious retroperitoneal or peritoneal perforation.

## Case presentation

**Figure 1:**
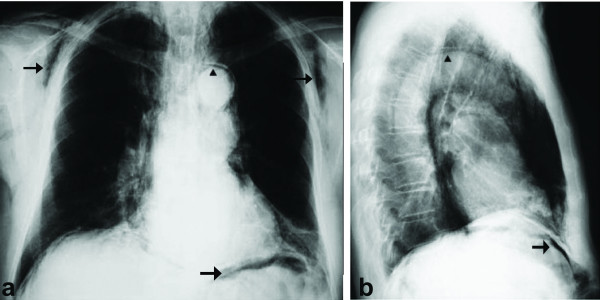
**Chest radiographs revealing the presence of subcutaneous emphysema, pneumomediastinum and pneumoperitoneum (arrows)**.

**Figure 2:**
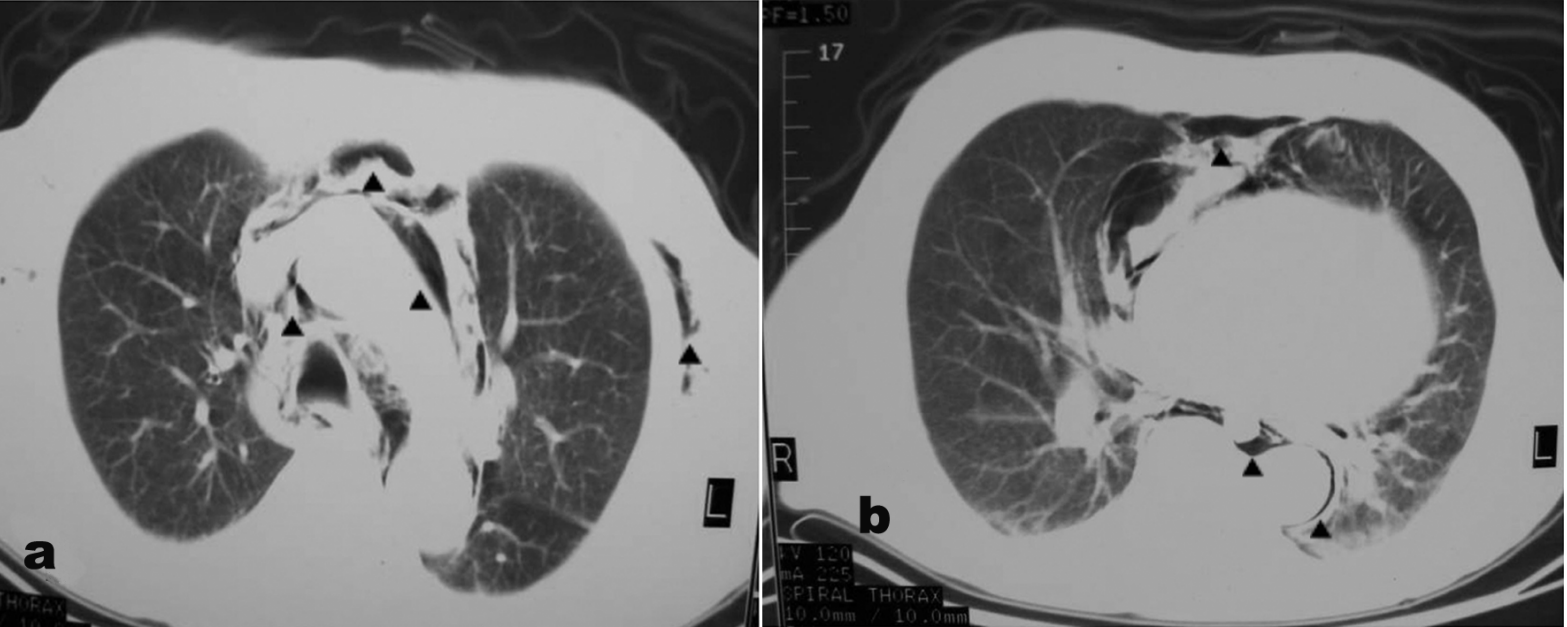
**Computed tomography of the chest indicating the presence of subcutaneous emphysema and pneumomediastinum (arrows)**.

A 78 year old Caucasian male referred to our department because of a history of recurrent jaundice and periodical appearance of right upper quadrant pain. The patient had undergone a post-Billroth II gastrectomy for peptic ulcer disease and cholecystectomy 48 and 7 years ago respectively. On admission, physical examination was unremarkable and laboratory evaluation showed no abnormalities with liver function tests being normal. Ultrasonography (U/S) of the liver revealed a common bile duct stone with no dilation of the common bile duct. Computed tomography (CT) showed the same findings as well. Magnetic resonance imaging (MRI) and magnetic resonance cholangiopancreatography (MRCP) revealed the existence of a common bile duct stone and confirmed the U/S and CT findings. The patient underwent an ERCP with the purpose to remove the offending stone and to perform endoscopic sphincterotomy. A long afferent limb of the Billroth II gastroenterostomy made locating the duodenal papilla technically difficult. After several attempts with no success, the procedure was abandoned for a few days later. The whole procedure had no obvious mediate complications. Immediately after the examination and for the rest of the day, there were no abnormal signs or symptoms. The morning after, the patient complained for crepitus in the soft tissues of the supraclavicular fossae and neck. Physical examination confirmed this finding. Plain radiographs of the chest and abdomen revealed subcutaneous emphysema, pneumomediastinum and pneumoperitoneum (Figure 1). An esophagogram with water-soluble contrast showed no evidence of perforation. Abdominal and chest CT-scan showed no evidence of retroperitoneal or peritoneal perforation but air was noted in the peritoneal cavity, the mediastinum and in the soft tissues of the neck (Figure 2). In the absence of revealing a site of free perforation, the patient was managed conservatively with gastric aspiration using a large-bore nasogastric tube, intravenous fluids and broad-spectrum antibiotics administration, and serial monitoring of hemodynamic parameters. After 48 hours of gastric aspiration, the patient started to drink fluids and he was on a light diet by the 5^th ^day after admission. Progressively, the signs of subcutaneous emphysema resolved. The patient was discharged on the 10^th ^hospital day without complains and without radiographic signs of pneumomediastinum and pneumoperitoneum.

## Discussion

The presence of pneumomediastinum implies that there is or have been a breach of an air-containing mediastinal structure. Air in the mediastinal tissues may originate from the respiratory tract, such as after blunt or penetrating trauma to the facial bones, pharynx, hypopharynx, trachea and main stem bronchi. Dental procedures – using compressed air – may result to facial and neck subcutaneous emphysema and pneumomediastinum. Severe straining, Valsalva maneuver and free perforation of the gastrointestinal tract may be responsible of the appearance of pneumomediastinum and subcutaneous emphysema [1].

In free perforation of the gastrointestinal tract, the air may enter from the peritoneal cavity to the mediastinum through the esophageal hiatus and the foramen of Morgagni. However, pneumomediastinum without evidence of perforation has been described after esophagogastroscopy [2], endoscopic sphincterotomy [1,3], sigmoidoscopy or colonoscopy [4-7], air contrast barium enema and endoscopic polypectomy [8,9]. There are also reports for pneumothorax complicating ERCP [10-12].

The most likely explanations for the simultaneous presence of pneumoperitoneum, pneumomediastinum and subcutaneous emphysema in our patient are: (a). The endoscopic tip injured the mucosa of the gastric mucosa or the long afferent limb of the Billroth II gastroenterostomy, allowing insufflated air to enter the wall and dissect along the perineural and perivascular sheaths to reach the mediastinum. (b). High pressure in the closed long afferent limb of the Billroth II gastroenterostomy may have contributed by forcing air through the mucosal break into the interstitial tissues.

Maunder et al described the anatomic route by which peritoneal air results in pneumomediastinum and pneumothoraces [13]. The soft tissue compartment of the neck, thorax and abdomen contains four regions defined as the subcutaneous tissue, prevertebral tissue, visceral space and previsceral space. The visceral space inverts the trachea and esophagus and continues with these structures into the mediastinum and bronchovascular sheaths. It follows the esophagus though the diaphragmatic hiatus into the retroperitoneal and peritoneal soft tissue space. Thus, there is continuity along the neck, thorax and abdomen. Air arising in any one of these regions could reach another area by "traveling" along the fascial planes.

Another explanation has been given by Kirschner, who described and categorized the clinical occurrence of peritoneopleural transphrenic passage of fluids or gases through, either congenital or acquired pores in the diaphragm as porous diaphragm syndromes [14].

Our patient experienced a mild retrosternal discomfort radiating into the neck. He did not experience respiratory difficulty. The physical examination can reveal crepitus; "Hamman's crunch" (crepitant sound heard at auscultation that varies with heartbeat), ecchymosis, masses, and erythema may be present in the face, neck, or torso. Typically, the history and physical examination are sufficient to determine the mechanism and timing of the injury and the patient's physiologic response.

The best tests to evaluate pneumomediastinum, pneumoperitoneum and subcutaneous emphysema are those that rapidly help to determine the location and size of perforation (if it exists), estimate the degree of contamination and help the clinician to develop a plan of treatment. A chest x-ray and plain abdominal x-rays define the findings, but because of being relatively insensitive, CT of the neck, chest and abdomen should be performed. CT can help to identify the source of mediastinal air. Contrast enhanced fluoroscopy of the pharynx and the esophagogastric region may help detecting the perforation. A water-soluble contrast study with diatrizoate meglumine (Gastrographin) is used to define an esophageal or gastric perforation, but a small leak or tear may seal spontaneously and be missed by this investigation in approximately 10% of the cases. If there is a high suspicion of perforation, a contrast study with dilute barium can detect smaller leaks [1]. Also, laryngoscopy, bronchoscopy and esophagoscopy may be useful to evaluate the damage and plan the management. Our patient underwent all the above examinations, except endoscopy, without being able to detect the site of perforation. Overall, with the above studies still there is a 5% to 10% risk that the perforation is present but not detected. If the clinical suspicion of perforation is high, the studies can be repeated after 12–24 hours, reducing the likelihood of a missed perforation to less than 1% to 2%.

When patients undergo evaluation for pneumomediastinum, the initial management is conservative. They should receive nothing by mouth; gastric aspiration and intravenous broad-spectrum antibiotics are appropriate. Total parenteral nutrition may be indicated. Repeated chest x-rays will document improvement and ensure that progression to pneumothorax will not be missed. If perforation is demonstrated in the imaging studies, surgical repair is indicated [1,13].

## Conclusion

Our case suggests that pneumomediastinum, pneumoperitoneum and subcutaneous emphysema without free esophageal perforation or perforation in the peritoneal cavity or the retroperitoneal space following endoscopy is benign, self-limited and need no surgical or radiological intervention.

## Competing interests

The authors declare that they have no competing interests.

## Authors' contributions

AK, ST and AG designed the study protocol; SN, KI and EN carried out the clinical assessment; ST, AK and AG drafted the manuscript. All authors read and approved the final manuscript. ST and AG are guarantors of the paper.

## Consent

We have obtained written, informed consent from the patient for open access publication of this case report. A copy of the written consent is available for review by the Editor-in-Chief of this journal.
